# Near‐Infrared‐Responsive Rare Earth Nanoparticles for Optical Imaging and Wireless Phototherapy

**DOI:** 10.1002/advs.202305308

**Published:** 2023-11-09

**Authors:** Pengye Du, Yi Wei, Yuan Liang, Ran An, Shuyu Liu, Pengpeng Lei, Hongjie Zhang

**Affiliations:** ^1^ State Key Laboratory of Rare Earth Resource Utilization Changchun Institute of Applied Chemistry Chinese Academy of Sciences Changchun Jilin 130022 China; ^2^ School of Applied Chemistry and Engineering University of Science and Technology of China Hefei Anhui 230026 China; ^3^ Ganjiang Innovation Academy Chinese Academy of Sciences Ganzhou Jiangxi 341000 China; ^4^ Department of Chemistry Tsinghua University Beijing 100084 China

**Keywords:** rare earth nanoparticles, near‐infrared light, optical imaging, photoconversion, phototherapy

## Abstract

Near‐infrared (NIR) light is well‐suited for the optical imaging and wireless phototherapy of malignant diseases because of its deep tissue penetration, low autofluorescence, weak tissue scattering, and non‐invasiveness. Rare earth nanoparticles (RENPs) are promising NIR‐responsive materials, owing to their excellent physical and chemical properties. The 4f electron subshell of lanthanides, the main group of rare earth elements, has rich energy‐level structures. This facilitates broad‐spectrum light‐to‐light conversion and the conversion of light to other forms of energy, such as thermal and chemical energies. In addition, the abundant loadable and modifiable sites on the surface offer favorable conditions for the functional expansion of RENPs. In this review, the authors systematically discuss the main processes and mechanisms underlying the response of RENPs to NIR light and summarize recent advances in their applications in optical imaging, photothermal therapy, photodynamic therapy, photoimmunotherapy, optogenetics, and light‐responsive drug release. Finally, the challenges and opportunities for the application of RENPs in optical imaging and wireless phototherapy under NIR activation are considered.

## Graphical abstract

In this review, the authors focus on rare earth nanoparticles (RENPs) as media for light conversion and discuss the photoconversion process under near‐infrared (NIR) excitation, as well as recent achievements in optical imaging, photothermal therapy, photodynamic therapy, photoimmunotherapy, optogenetics, and light‐responsive drug release. Finally, the opportunities and challenges of NIR‐responsive RENPs for optical imaging and wireless phototherapy are discussed.

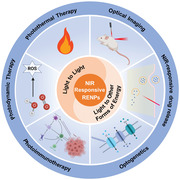

## 1 Introduction

As modern society continues to evolve, a sharp disparity emerges between the rising demands of public health and the low efficacy and severe side effects of the traditional treatment methods (e.g., surgery, chemotherapy, and radiotherapy) used for major diseases, particularly cancer.^[^ ^1^, ^2^, ^3^ ^]^ In light of the above, effective and robust treatment strategies and drugs must be developed to achieve optimal therapeutic effects.^[^ ^4^, ^5^, ^6^ ^]^ Nanomaterials are considered one of the most promising candidates for addressing this issue, owing to their controlled synthesis, easy surface modification and functionalization, and long in vivo circulation time.^[^ ^7^, ^8^, ^9^ ^]^ Efficient cancer therapy is often achieved using external stimuli.^[^ ^10^, ^11^ ^]^ The use of light as an external stimulus offers the advantages of spatiotemporal selectivity, non‐invasiveness, and few side effects, rendering it one of the most promising alternatives.^[^ ^12^, ^13^, ^14^, ^15^ ^]^ Of the numerous light‐responsive bioprobes, rare earth nanoparticles (RENPs) have attracted considerable attention because of their excellent physical and chemical properties.^[^ ^16^, ^17^, ^18^, ^19^ ^]^

**Figure 1:**
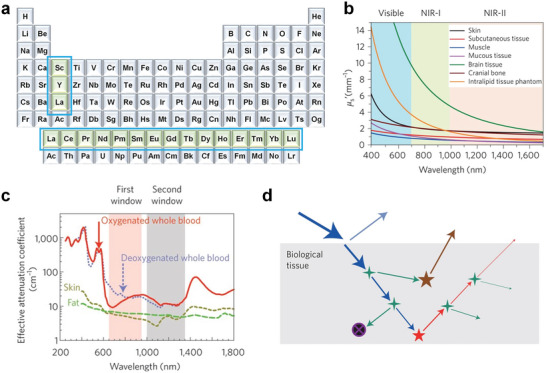
Periodic table of elements and scattering of different wavelengths of light. a) Location of rare earth elements in the periodic table (blue rectangle). b) Scattering coefficients of different biological tissues and Intralipid scattering tissue phantom as a function of wavelength in the 400–1700 nm region. c) The effective attenuation coefficients of absorption and scattering from oxygenated blood, deoxygenated blood, skin, and fatty tissues. The latter two exhibit the lowest coefficients in both NIR‐I (pink shaded area) or NIR‐II (grey) windows. Reproduced with permission.^[^ ^37^ ^]^ Copyright 2009, Springer Nature. d) Light‐tissue interactions resulting from impinging excitation light (blue), interface reflection (cyan), scattering (green), absorption (black circle with purple cross), and autofluorescence (brown), all of which contribute to the loss of signal (fluorescence, red) and increase of noise. b,d) Reproduced with permission.^[^ ^36^ ^]^ Copyright 2017, Springer Nature.

In 1794, the Finnish chemist John Gadolin isolated the element Y from a piece of heavy ore‐shaped pitch, which was the first recorded discovery of rare earth elements.^[^ ^20^ ^]^ Rare earth elements were grouped together even before their properties were fully understood, because of their similar extra‐nuclear electronic structures and chemical properties and predominant appearance in the Earth's crust in the form of oxides. Rare earth elements consist of Sc, Y, and lanthanides (Ln), which is a group of elements occupying the third group and sixth period of the periodic table (namely, La, Ce, Pr, Nd, Pm, Sm, Eu, Gd, Tb, Dy, Ho, Er, Tm, Yb, and Lu) (**Figure** **1**a).^[^ ^5^, ^21^, ^22^, ^23^ ^]^ The electronic configuration of Ln is of the form [Xe] 4f^0–14^5s^2^5p^6^5d^0–1^6s^2^. The 4f electronic configuration of rare earth ions has 1639 energy levels, and the number of transitions between them can be as high as 199 177. The rich energy‐level structure of the 4f subshell provides abundant opportunities for the conversion of light from one wavelength to another or to other forms of energy, such as thermal and chemical energies.^[^ ^6^, ^24^, ^25^ ^]^ The 4f–4f transition of lanthanide ions (Ln^3+^) is less affected by external crystalline or coordination fields, owing to the shielding effect of the 5s^2^5p^6^ electrons; hence, lanthanides exhibit unique properties compared to conventional optical probes such as organic dyes, quantum dots, and carbon nanotubes.^[^ ^26^, ^27^, ^28^ ^]^ RENP luminescence often exhibits long luminescence lifetimes in the scale of milliseconds. Further, it offers the benefits of large Stokes or anti‐Stokes shifts, low toxicity, excellent chemical stability, good biocompatibility, deep tissue penetration, and high resistance to photobleaching and scintillation.^[^ ^24^, ^29^, ^30^, ^31^ ^]^ The rich energy level structure of rare earth ions can also play a crucial role in converting light to other forms of energy. The transition of electrons from high‐ to low‐energy levels is accompanied by the release of energy. Radiation‐free transition is often accompanied by the generation of heat, that is, the photonic energy is converted to heat energy. This energy conversion can be regulated to some extent by controlling the type and concentration of dopant ions. Photochemical reactions can be triggered to produce active species for wireless phototherapy either through electron transfer or energy transfer with suitable substrates.^[^ ^32^, ^33^ ^]^

The wavelength of light is also a crucial parameter for in vivo biological applications. For the vast majority of biological tissues, the degree of photon scattering is inversely proportional to approximately λ^α^, where λ is the photon wavelength and α ranges from 0.2 to 4. Moreover, the autofluorescence of biological tissues decreases with increasing wavelength (Figure 1b,c).^[^ ^34^, ^35^, ^36^, ^37^ ^]^ Fortunately, RENPs are highly efficient in responding to long‐wavelength near‐infrared (NIR) light. The above features make RENPs well‐suited for phototherapy against malignant diseases. As a comprehensive class of nanoplatforms, RENPs have tunable morphology and surface properties and abundant surface modification sites, allowing the integration of multiple diagnostic and therapeutic functions into a single carrier via loading, chemical coupling, or integration to construct comprehensive phototherapeutic platforms.^[^ ^38^, ^39^, ^40^, ^41^ ^]^

In this review, we focus on the functionality of RENPs as light conversion media and systematically discuss their photoconversion process under NIR excitation, as well as the recent achievements in optical imaging, photothermal therapy (PTT), photodynamic therapy (PDT), photoimmunotherapy (PIT), optogenetics, and light‐responsive drug release. The opportunities and challenges that coexist in this promising field are also presented in detail. Finally, we provide our insights on the prospects and future directions in applying NIR‐responsive RENPs in optical imaging and wireless phototherapy.

## 2 NIR‐Responsive RENPs for Photoconversion

Light irradiation is the basis of optical imaging and wireless phototherapy. However, biological media can absorb photons, and biological tissues cause photon scattering, which leads to low tissue penetration in the visible window. Interference from autofluorescence is also observed in biological tissues (Figure 1d).^[^ ^36^, ^42^, ^43^ ^]^ These problems can be addressed using NIR light. NIR light causes weak autofluorescence, low tissue absorption and scattering, and deep tissue penetration, which renders it well‐suited for biological applications.^[^ ^44^, ^45^, ^46^ ^]^ As nanomaterials with photoconversion functions, RENPs are among the most promising optical probes because of their unique properties such as narrow‐band emission and photostability.^[^ ^38^, ^39^, ^47^ ^]^ Light‐to‐light conversion by RENPs is mainly of three types: upconversion, downshifting, and downconversion. Moreover, the conversion of light energy to other forms of energy using RENPs is also gaining widespread attention from researchers, which is briefly described later in this section.^[^ ^4^, ^48^ ^]^

### 2.1 Light‐to‐Light Conversion

Light‐to‐light conversion is the most well‐known form of photoconversion conducted using NIR‐responsive RENPs. It is also one of the most well‐studied areas into which considerable efforts have been invested. In this section, we focus on the three most common luminescence phenomena: upconversion, downshifting, and downconversion. Each phenomenon is discussed in terms of its most plausible principles and its major NIR response systems. Additionally, we discuss a special luminescence phenomenon exhibited by RENPs, namely persistent luminescence (PL).

#### 2.1.1 Upconversion Luminescence

Upconversion luminescence (UCL) is a process in which low‐energy photon (e.g., NIR light) excitation results in the emission of high‐energy photons (e.g., UV and visible light) through continuous multi‐photon absorption and energy transfer.^[^ ^49^, ^50^, ^51^ ^]^ The luminescence of rare earth upconversion nanoparticles (UCNPs) offers advantages such as narrow emission spectral bands, long lifetimes, large anti‐Stokes shifts, and good photostability.^[^ ^10^, ^31^ ^]^ In contrast to direct irradiation with short‐wavelength light sources, converting NIR light using UCNPs allows for deep penetration, high spatial resolution, and low autofluorescence properties.^[^ ^52^, ^53^ ^]^

**Figure 2:**
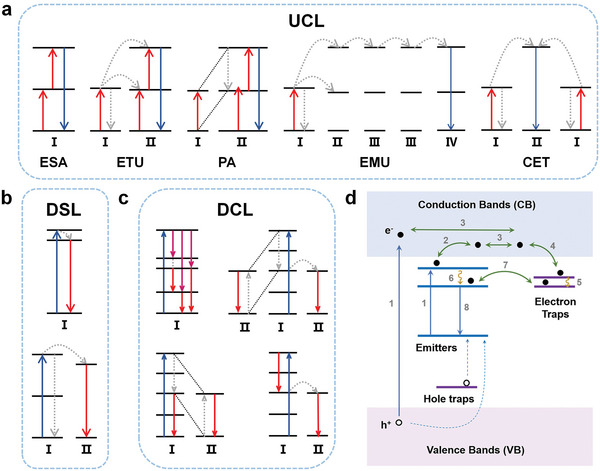
Schematic illustration of possible mechanisms for different kinds of light‐to‐light conversions and PL. a) UCL categories: ESA, ETU, PA, EMU, and CET, b) DSL, c) DCL, and d) PL. Reproduced with permission.^[^ ^80^ ^]^ Copyright 2023, Wiley‐VCH.

Several studies have been conducted to elucidate the UCL mechanism. Currently, UCL mechanisms can be roughly divided into five categories: excited state absorption (ESA), energy transfer upconversion (ETU), photon avalanche (PA), energy migration‐mediated upconversion (EMU), and cooperative energy transfer (CET) (**Figure** **2**a).^[^ ^54^, ^55^ ^]^

ESA is the most common mechanism of UCL. It is characterized by the transition of an activator ion from the ground‐state energy level to a higher energy level through successive multiphoton absorption, culminating in the emission of a high‐energy photon.^[^ ^56^, ^57^ ^]^ Under these conditions, the intensity of UCL is proportional to the nth power of the pump power, where n is the number of pump photons absorbed by the activator ion during the excitation process.

The type of ETU closely depends on the type of energy transfer, which can occur between the same or different ions. In a typical ETU upconversion system, a sensitizer ion is excited by an external light source.^[^ ^55^, ^58^, ^59^ ^]^ Upon interacting with an activator ion that meets the energy‐matching requirements, the sensitizer ion transfers its energy to the activator ion, which excites the activator ion to a higher energy level. The sensitizer ion then returns to the ground state in a radiation‐free relaxation mode. The excited activator ion can receive additional energy to transition to a higher energy level. This type of ETU is known as a successive energy transfer.^[^ ^50^, ^60^ ^]^ The energy transfer between two ions that are simultaneously at an excited state, or between two energy levels of the same ion, is called cross‐relaxation (CR).^[^ ^61^, ^62^ ^]^ In many UCL materials, the ETU and ESA mechanisms coexist and the upconversion is realized via their collaboration. Moreover, to compensate for the energy mismatch during energy transfer, this process allows phonons to participate. Similar to the ESA, the intensity of ETU‐driven luminescence is usually proportional to the square or nth power of the pump power.

PA does not occur on most occasions, owing to its apparent power dependence. The concept of PA was first introduced by Chivian et al.^[^ ^63^ ^]^ Specifically, the CR between two energy levels of the same ion results in the occupation of the intermediate excited energy levels of other ions, and the population in these levels increases like an avalanche, which gives it the name PA.^[^ ^64^, ^65^, ^66^ ^]^ However, achieving PA comes with several challenges. The pumping wavelength for an ion corresponds to the energy gap between one of its excited states and its upper energy level, rather than the energy gap between its ground‐state energy level and the excited state. PA exhibits a distinct dependence on the pump power; only a weak UCL is exhibited below the pump‐power threshold and the UCL intensity significantly increases above the threshold, where the pump light is strongly absorbed. The PA process depends on the accumulation of ions in the excited state. Therefore, significant PA may occur only when the ion doping concentration is sufficiently high.

Compared to the above mechanisms, EMU involves two additional components: accumulators and migrators. Sensitizers are first excited at a suitable light wavelength. They transfer their energy to accumulator ions, which excite these ions to a higher energy level. The migrators then capture the energy transferred from the accumulators and ultimately transfer the energy to the activator ions. Migrators play a crucial role in ensuring long‐distance energy transfer in EMU, particularly in core–multi‐shell structures.^[^ ^67^, ^68^ ^]^

CET occurs only in a few upconversion systems.^[^ ^69^, ^70^ ^]^ This process can be considered an interaction between three ions. In particular, simultaneous energy transfer from two ions in an excited state to a ground‐state ion causes the latter to transition to a higher energy level. Unlike in conventional ETU, the activator ions in CET achieve UCL with the help of a virtual intermediate energy level. It is an uncommon UCL mechanism because it is difficult to achieve.

#### 2.1.2 Downshifting Luminescence

Downshifting emission is the predominant luminescence process observed in most photoluminescent materials. In this process, the wavelength and energy of the emitted light are higher and lower than those of the excitation light, respectively. This phenomenon was discovered by Stokes in 1852.^[^ ^24^, ^71^, ^72^ ^]^ In a typical downshifting process, the excitation‐light source transfers photon energy to a rare earth ion, causing the latter to transition from the ground state to an excited state. Subsequently, the ion transitions to a lower energy level, which causes the emission of a low‐energy photon and induces the transition of the ion back to the ground state. Theoretically, the number of photons remains constant throughout the process; however, the photon energy decreases (Figure 2b). Therefore, the theoretical quantum efficiency is less than 100%.

The downshifting luminescence (DSL) of RENPs activated by NIR radiation is of considerable interest to researchers because both excitation and emission occur in the NIR region. In 2006, Wang et al. proposed a simple method to synthesize highly water‐soluble LaF_3_ nanocrystals directly in an aqueous solution without using any ligands. The crystals doped with Nd^3+^ exhibited downshifting emissions under 802 nm excitation.^[^ ^73^ ^]^ Yb^3+^ DSL can be achieved through Nd^3+^ sensitization.^[^ ^74^ ^]^ Riman et al. synthesized NaYF_4_:Yb^3+^, Ln^3+^ (Ho^3+^, Tm^3+^, and Pr^3+^) nanoparticles and compared their optical efficiencies with that of NaYF_4_:Yb^3+^, Er^3+^ under 980 nm excitation. The efficiencies were ranked in the order Er^3+^ > Ho^3+^ > Tm^3+^ > Pr^3+^.^[^ ^75^ ^]^ Their study implied that Er^3+^ may be the most promising candidate for optical imaging. As Ln^3+^ ions have relatively strict energy‐level limits for photon absorption and emission, the rare earth activator ions currently used for DSL in NIR‐light response are mainly Nd^3+^ (^4^F_3/2_ → ^4^I_11/2_, ^4^F_3/2_ → ^4^I_13/2_), Yb^3+^ (^2^F_5/2_ → ^2^F_7/2_), Er^3+^ (^4^I_13/2_ →^4^I_15/2_), Ho^3+^ (^5^I_6_ → ^5^I_8_), Tm^3+^ (^3^H_4_ → ^3^F_4_), and Pr^3+^ (^1^G_4_ → ^3^H_5_).^[^ ^72^, ^74^ ^]^

#### 2.1.3 Downconversion Luminescence

Downconversion Luminescence (DCL) is a process opposite to upconversion and refers to the phenomenon of absorbing one high‐energy photon and emitting two or more low‐energy photons; therefore, it is also known as quantum cutting.^[^ ^76^ ^]^ Dexter argued that quantum efficiencies exceeding 100% could be obtained if two acceptors simultaneously receive half of the energy from the same donor, a concept he developed in 1957.^[^ ^77^ ^]^ The first experimental report on this phenomenon was published in 1974 when Piper et al. investigated photon cascade emission from a YF_3_:Pr^3+^ system and experimentally observed the quantum cutting event. Under vacuum UV excitation in the Hg resonance line at 185 nm, Pr^3+^ transitions from the ground state to the 5d energy level and then relaxes to the ^1^S_0_ energy level. The first photon, with a wavelength of 400 nm, is emitted through the ^1^S_0_ → ^3^P_2_,^1^I_6_ transition. Subsequently, from ^1^I_6_ relaxation to ^3^P_0_ energy level, the second visible photon is radiated through the ^3^P_0_ → ^3^F_J_,^3^H_J_ transition. The quantum efficiency of this system can reach 140% because of the quantum cutting effect.

DCL can be performed using either a single ion or two ions (Figure 2c).^[^ ^78^ ^]^ A single rare earth ion absorbs a high‐energy photon and gets excited to a higher energy level. Subsequently, it transitions to other lower energy levels, releasing photons of different wavelengths. When two rare earth ions interact with each other, one of them acts as a sensitizer and the other acts as an activator. The sensitizer is excited to a higher energy level by an exogenous light source, and CR can occur between it and the activator, causing the activator ion to transition from the ground state to an excited state. Subsequently, the activator ion returns to the ground state via radiative transitions and emits low‐energy photons. The sensitizer returns to a lower‐energy excited state, at which point two possibilities emerge: either the sensitizer transfers energy to a neighboring activator ion again, repeating the previous step, or it undergoes a direct radiative transition from this excited state back to the ground state, accompanied by photon emission. Moreover, the sensitizer can emit a low‐energy photon upon direct transition back to a lower excited state, followed by energy transfer with the activator ion, which emits the second photon.

Numerous materials have been reported to exhibit UV‐blue‐activated rare earth quantum cutting. By contrast, only a few materials exhibit DCL under NIR excitation. Tm^3+^ is a promising alternative. Yu et al. described the quantum cutting of 790 nm NIR‐excited Tm^3+^‐doped phosphor.^[^ ^79^ ^]^ Tm^3+^ is excited from the ^3^H_6_ ground state to the excited state ^3^H_4_. Subsequently, it radiatively transitions to the ^3^H_5_ state, emitting an NIR photon at a wavelength of 1460 nm. This is followed by a radiative transition back to the ground state, emitting a second photon at 1800 nm. Unfortunately, NIR‐activated quantum‐cutting materials have rarely been used in biological applications. Its potential may become buried, an outcome researchers are keen to avoid, therefore necessitating further in‐depth investigations in this field.

#### 2.1.4 Persistent Luminescence (PL)

PL materials are optical materials that, when irradiated by an external energy source for a period, exhibit luminescence even after the cessation of the irradiation. The luminescence can last minutes, hours, or even days.^[^ ^80^, ^81^ ^]^ In a conventional PL process (Figure 2d), after a sufficiently long period of energy irradiation, electrons in the valence band (VB) or ground state of the emitter are promoted to the conduction band (CB), where they move freely (processes 1, 2, and 3). Electron traps capture the CB electrons and store them for a period (process 4), whereas nonradiative transitions in the trap energy level may generate deeper electron traps (process 5). A few electrons in the excited state of the emitter may also be captured by the energy‐matched traps (processes 6 and 7). This process is known as the “charging” of the materials. The storage capacity of the trap centers strongly depends on the number of electrons as well as the concentration and depth (the energy required to release electrons from the traps) of the traps. When the stored electrons are physically stimulated, they gradually escape from the traps, return to a lower excited state, and radiatively transition back to the ground state to emit photons (process 8). This process is known as “discharging.” The charging and discharging processes can be repeated until the instability limit of the PL material is reached.

The first documented account of the PL phenomenon was recorded in the early 17^th^ century when the Italian alchemist Vincenzo Cascariolo accidentally obtained some stones that glowed red at night during the process of “making gold from stone.”^[^ ^82^ ^]^ The stones contained BaSO_4_, which is partially reduced to BaS when heated at high temperatures. BaS combines with Cu to produce BaS:Cu^2+^, which exhibits PL. In 1866, Sidot synthesized the first PL material, ZnS:Cu^2+^, which initiated research on sulfide‐system PL materials, constituting the first generation of PL materials.^[^ ^83^ ^]^ In the 1960s, Palilla discovered PL in the aluminate material SrAl_2_O_4_:Eu^2+^, which exhibits superior PL properties compared to conventional sulfides. Since then, rare earth PL materials have flourished and become pivotal in the field of PL research.^[^ ^84^ ^]^ In 2014, Pan et al. presented the novel concept of upconversion persistent luminescence (UCPL).^[^ ^85^ ^]^ They doped the PL phosphor Zn_3_Ga_2_GeO_8_:1%Cr^3+^ with the common upconversion sensitizer/activator ion pair Yb^3+^/Er^3+^ and realized PL emission at 700 nm under 980 nm excitation, with an afterglow lasting more than 24 h. This study extends the roster of PL power sources from X‐rays, UV light, and visible light to include NIR. Similar tri‐doped systems have been obtained for UCPL in the host matrices Zn_3_Ga_2_SnO_8_,^[^ ^86^ ^]^ Zn_1.3_Ga_1.4_Sn_0.3_O_4_,^[^ ^87^ ^]^ and ZnGa_2_O_4_.^[^ ^88^ ^]^ Sengar et al. realized UCPL in Gd_3_Al_2_Ga_3_O_12_:Ce^3+^, Cr^3+^ through the CET mechanism.^[^ ^89^ ^]^ It can be excited by two 800 nm photons and exhibits multiphoton emission in the visible and NIR regions, indicating potential for bioimaging applications. Zhou et al. prepared a broadband‐responsive NIR PL phosphor, NaScGe_2_O_6_:Cr^3+^, which can be effectively excited by an 808 nm NIR light source.^[^ ^90^ ^]^ The application of this material in wearable NIR biosensors offers innovative insights and fresh perspectives on the use of PL materials as biomarkers and biosensors.

### 2.2 Conversion of Light to Other Forms of Energy

It is well known that energy can be transferred not only in the same form but also between different forms. Light energy can be converted into various forms such as thermal and chemical energies. Photothermal materials can efficiently convert the energy of absorbed light into heat, thereby changing the temperatures of the materials and their surroundings. Owing to these properties, they are often used as photothermal agents. In addition, photodynamic materials can utilize the absorbed light to induce photochemical reactions. Fortunately, RENPs are remarkably well‐suited for efficiently converting light into multiple energy forms owing to their unique physicochemical properties. NIR‐responsive RENPs are essential in wireless phototherapy.^[^ ^32^, ^33^, ^91^ ^]^ The design, synthesis, and utilization of RENPs with diverse compositions and functions and the selection of suitable light sources for external field stimulation are crucial to achieving highly efficient, controllable, low‐toxic, and side‐effect‐free treatment of malignant diseases and promoting development in this field.

## 3 Application of NIR‐Responsive RENPs

Owing to excellent photoconversion properties, RENPs can be used for various light‐mediated applications. Recent studies have confirmed that the use of NIR light in biological applications offers extraordinary advantages over visible and UV light; therefore, NIR‐responsive RENPs with low toxicity and good photostability have been widely studied, and their potential applications are worth further exploration. In this section, we focus on optical imaging, PTT, PDT, PIT, optogenetics, and light‐responsive drug release systems realized using the energy conversion accomplished by NIR‐responsive RENPs.

### 3.1 Optical Imaging

Generally, drugs or surgical methods that are effective against early‐stage diseases are not applicable to late‐stage diseases. Therefore, the diseases must be precisely diagnosed as early as possible to maximize therapeutic efficacy. Currently, diagnostics primarily involve the targeted imaging of cells and tissues, as well as the capture, isolation, and ultrasensitive testing of proteins and genetic biomarkers in the blood.^[^ ^92^ ^]^ Compared to other existing imaging techniques, optical imaging has the advantages of having low cost, fast feedback, high sensitivity, and no radiation hazards. Therefore, it has attracted considerable attention in recent years.^[^ ^42^, ^93^, ^94^, ^95^, ^96^ ^]^ In this section, a brief introduction to optical imaging based on the light‐to‐light conversion by RENPs under NIR activation is provided. Specifically, the discussion focuses on UCL, DSL, and PL imaging. DCL imaging is not discussed in this section owing to the limited reach of its applications.

#### 3.1.1 Upconversion Luminescence Imaging

**Figure 3:**
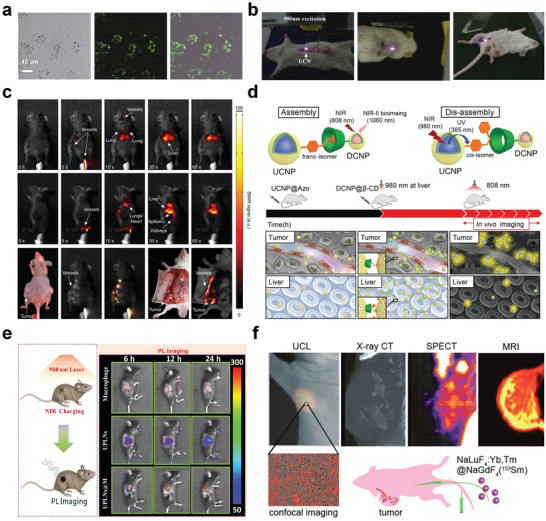
NIR‐responsive RENPs for optical imaging. a) Bright field, confocal, and superimposed images of live human colonic adenocarcinoma cells (HT29), with UCNPs attached. b) In vivo imaging of rats injected with UCNPs below abdominal skin (left), thigh muscles (middle), or below the skin of the back (right). a,b) Reproduced with permission.^[^ ^100^ ^]^ Copyright 2008, Elsevier. c) Real‐time video capture of the biodistribution of intravenously injected downshifting RENPs in hairless mice using the imaging system prototype: ventral (top) and left lateral (middle) views. Nude mice bearing melanoma xenografts were intravenously injected with LDNPs and imaged near surrounding tumor regions before dissection (bottom) from the ventral aspect. Reproduced with permission.^[^ ^47^ ^]^ Copyright 2013, Springer Nature. d) Supramolecular recognition‐induced assembly and 980 nm NIR‐regulated disassembly of nanoparticles (top); In vivo assembly of UCNP@Azo (the first injection) and DCNP@β‐CD (the second injection) with improved tumor targeting, two‐staged in‐sequence injection strategy, and 980 nm NIR‐regulated in vivo disassembly with rapid clearance in the liver (bottom). Reproduced with permission.^[^ ^112^ ^]^ Copyright 2018, Wiley‐VCH. e) The schematic diagram of NIR‐active upconverting PL nanophosphors (UPLNs) used for long‐time PL imaging (left) and in vivo PL imaging of different treatment groups, namely the macrophages, UPLNs, and UPLN‐loaded macrophages (UPLNs@M) groups, at different times (right). Reproduced with permission.^[^ ^120^ ^]^ Copyright 2018, American Chemical Society. f) The bioimaging application of NaLuF_4_:Yb^3+^, Tm^3+^@NaGdF_4_(^153^Sm). Reproduced with permission.^[^ ^122^ ^]^ Copyright 2013, American Chemical Society.

The absence of bleaching and scintillation, coupled with a high signal‐to‐noise ratio and low toxicity, renders RENPs that undergo upconversion under NIR activation promising contrast agents for fluorescence imaging, and they are widely used in cellular or in vivo imaging.^[^ ^97^ ^]^ Cellular imaging provides an intuitive visualization of physiological processes at the cellular or subcellular level, whereas in vivo fluorescence imaging can be used to detect morphological, anatomical, and physiological anomalies in tissues at a subcellular resolution.^[^ ^98^, ^99^ ^]^ The application of UCNPs for the fluorescence imaging of cells and living bodies was first reported by Zhang et al. in 2008, opening new vistas for RENP applications (**Figure** **3**a,b).^[^ ^100^ ^]^ Compared to blue and green lights, red light can reduce light scattering, absorbance, and autofluorescence in tissues owing to the lack of effective endogenous red‐light absorbers. Zhang et al. modulated the monochromatic upconversion emission of red light under NIR excitation such that both the emission and excitation wavelengths were within the “tissue optical window,” which renders the system well‐suited for deep tissue imaging.^[^ ^101^, ^102^ ^]^ Chen et al. utilized the ^3^H_5_ energy level of Tm^3+^ to modulate energy transfer between Er^3+^ ions, resulting in bright red‐light emission from NaErF_4_:0.5%Tm^3+^@NaYF_4_ nanoparticles under three excitation wavelengths: 808, 980, and 1532 nm.^[^ ^103^ ^]^ The application of three different light sources for in vivo imaging allowed the precise management of excitation and emission in a wide optical window, enabling the consideration of optimal detection sensitivity, light penetration, and photothermal effects. Several UCNPs reported by our group have been systematically studied for fluorescence imaging applications.^[^ ^104^, ^105^, ^106^ ^]^ In addition, UCL has been applied in biodistribution imaging,^[^ ^107^ ^]^ target imaging,^[^ ^108^ ^]^ lymphatic imaging,^[^ ^109^ ^]^ vascular imaging,^[^ ^110^ ^]^ and cellular tracer imaging,^[^ ^111^ ^]^ guiding researchers to investigate further in this field.

#### 3.1.2 Downshifting Luminescence Imaging

The RENPs that undergo DSL under NIR activation have been mainly used for NIR‐II imaging. This is an emerging field, only approximately a decade old; it was pioneered in 2013 when Naczynski et al. realized multispectral and in vivo real‐time NIR‐II imaging using NaYF_4_:Yb^3+^, Er^3+^ (Figure 3c).^[^ ^47^ ^]^ However, the development of NIR‐II imaging has been rapid. It exhibits better performance in terms of background interference, penetration depth, and spatiotemporal resolution, which are crucial in bioimaging.^[^ ^4^, ^42^ ^]^ Zhang et al. assembled and disassembled azobenzene (azo)‐modified UCNPs and β‐cyclodextrin (β‐CD)‐modified downshifting nanoprobes according to host–guest interactions to realize precise in vivo imaging of tumor cells (Figure 3d).^[^ ^112^ ^]^ They obtained 1060 nm emissions from Nd^3+^ under 808 nm excitation and used the emissions for NIR‐II imaging, which exhibited a high resolution (< 10 µm) and signal‐to‐noise ratio (≈15). Correspondingly, the UV and visible emissions from Tm^3+^ ions excited by 980 nm NIR light triggered an isomerization transition of azo between its trans and cis forms, which in turn controlled the in vivo assembly and disassembly of the azo. In addition, this supramolecular self‐assembly quadrupled the accumulation of RENPs within the tumor, extended the retention time to 5 h, and improved the clarity in imaging the reticuloendothelial tissue to reduce liver accumulation and potential long‐term biotoxicity. Chang et al. demonstrated the potential of Er^3+^‐doped LiTmF_4_ nanoparticles for in vivo NIR‐IIc (1700–2000 nm) imaging applications.^[^ ^113^ ^]^ Er^3+^ acts as an energy relay station: its ^4^I_11/2_ and ^4^I_13/2_ energy levels assist in the energy transfer of Tm^3+^ and enhance the NIR‐IIc emissions at 1800 nm. This system can be excited by four wavelengths, 808, 980, 1208, and 1530 nm, with all producing bright 1800 nm emissions. Undoped LiTmF_4_ exhibits two‐photon emissions at 1450 and 1800 nm upon 800 nm excitation via quantum cutting. This system may be considered a prototype for the application of NIR‐responsive DCL materials in bioimaging. In addition, NIR‐II probes based on RENPs have been used for the in vivo imaging of biological tissues, such as those of the vascular system,^[^ ^114^ ^]^ brain,^[^ ^115^ ^]^ tumor organs,^[^ ^112^ ^]^ visceral organs,^[^ ^116^ ^]^ heat‐producing adipose tissues,^[^ ^117^ ^]^ and bones,^[^ ^118^ ^]^ providing precise information.

#### 3.1.3 PL Imaging

Rare earth PL materials can be excited before using them for in vivo detection and imaging, enabling the sensing and imaging of living organisms in the absence of excitation. This can effectively prevent background interference from continuous excitation. By contrast, NIR excitation allows nanoparticles to be well‐excited even in vivo, which satisfies the requirement for precise real‐time imaging guidance. The use of NIR‐responsive PL materials for bioimaging applications was first reported by Xue et al., who applied the UCPL materials Zn_3_Ga_2_GeO_8_:Cr^3+^, Yb^3+^, Er^3+^ for in vivo imaging.^[^ ^86^ ^]^ Han et al. physically mixed UCNPs with CaS:Eu^3+^, Tm^3+^, Ce^3+^ PL nanoparticles to form a thin film using polymethyl methacrylate. The UCNPs were excited using 980 nm laser light, which resulted in the emission of green light. Subsequently, the PL nanoparticles were induced to produce a red afterglow.^[^ ^119^ ^]^ Small pieces of the film (1 × 2 × 2 mm^3^) were implanted into the legs of mice to mimic biological implants. The film could be excited using a 980 nm light at any time; however, constant radiation was not required, which avoids the risk of laser‐induced overheating. This physical mixture can be transformed into a heterostructure consisting of a combination of two nanoparticles, which further enhances the bioimaging effect and offers the possibility of realizing more comprehensive optical and biological imaging. Zheng et al. performed in vivo tracking and labeling of tumor therapeutic macrophages using Zn_2_SiO_4_:Mn^3+^, Y^3+^, Yb^3+^, Tm^3+^ UPCL phosphors, which were used to localize the distribution of these macrophages via whole‐animal optical‐imaging. They successfully accomplished real‐time precision imaging‐mediated cell therapy of tumors (Figure 3e).^[^ ^120^ ^]^ Currently, relatively few NIR‐responsive PL materials are used in biological applications. The depth of exploration in this area is insufficient, suggesting a promising future direction.

#### 3.1.4 Combined Optical Imaging with Other Imaging Modalities

Owing to the complexity of biological tissues, optical imaging often suffers from interference. Other imaging modalities have limitations and cannot always be fully utilized.^[^ ^121^ ^]^ This necessitates the synergization of multiple imaging modalities. Rare earth ions can easily replace each other in their lattice positions in nanomaterials because of their similar electronic structures and chemical properties; therefore, the above problem can be addressed using materials doped with multiple rare earth ions. Ln elements exhibit potential X‐ray attenuation properties and are well‐suited for computed tomography (CT) owing to their heavy‐atom properties.^[^ ^122^ ^]^ Ln^3+^ containing unpaired electrons can theoretically be used in magnetic resonance imaging (MRI).^[^ ^122^ ^]^ Radioactive ^177^Lu and ^153^Sm, which have half‐lives of 6.7 days and 46.3 h, respectively, can emit γ‐rays for single‐photon emission computed tomography (SPECT).^[^ ^123^, ^124^ ^]^ The isotope ^86^Y, which is a common nuclide used in positron‐emission computed tomography, has a half‐life of only 14.7 h.^[^ ^125^ ^]^ Yu et al. synthesized an Nd^3+^‐doped NaGd(WO_4_)_2_ nanostructure decorated with a hydrophilic layer for realizing NIR‐II/MRI/CT trimodality imaging under 808 nm excitation.^[^ ^124^ ^]^ Sun et al. considered the function of every rare earth ion in bioimaging and designed a core‐shell NaLuF_4_:Yb^3+^, Tm^3+^@NaGdF_4_:^153^Sm^3+^ contrast agent, realizing a single multifunctional nanoplatform incorporating the functions of UCL, CT, MRI, and SPECT imaging (Figure 3f).^[^ ^122^ ^]^

Modern treatments require rapid, accurate, and comprehensive pathophysiological information for diagnosis. Multimodal imaging addresses this requirement by incorporating the advantages of different imaging tools. Furthermore, the integration of multiple imaging techniques highlights the significant potential of RENPs for precise diagnosis of different types of cancers.

### 3.2 Photothermal Therapy

#### 3.2.1 Mechanism of PTT

PTT utilizes materials with high photothermal conversion efficiency (photothermal agents) to convert light energy into heat energy, causing irreversible overheating damage to tumor tissues.^[^ ^126^, ^127^, ^128^ ^]^ PTT has the advantages of high efficacy, low drug resistance, and negligible systemic toxicity. Photothermal agents accumulate within the tumor tissues to absorb light and convert it into thermal energy to achieve local ablation of solid tumors. It is a minimally or non‐invasive technique and has fewer side effects in normal tissues.^[^ ^129^, ^130^, ^131^ ^]^ Recently reported photothermal agents exhibiting excellent performance mainly include carbon‐based nanomaterials, noble metal/transition metal‐based materials, organic dyes, metal/covalent/hydrogen‐bonded organic frameworks, and polymerized nanoparticles. RENPs possess an excellent photoconversion ability and rich spectral structure, which suggest the potential of using them as both independent photothermal agents and functional nanocomposites constructed in combination with other types of photothermal agents.^[^ ^132^, ^133^, ^134^ ^]^ Depending on the types and concentrations of sensitizers and activators, a wide spectral range from UV to NIR can be used for NIR excitation to induce efficient PTT.

#### 3.2.2 NIR‐Responsive RENPs as Photothermal Agents

**Figure 4:**
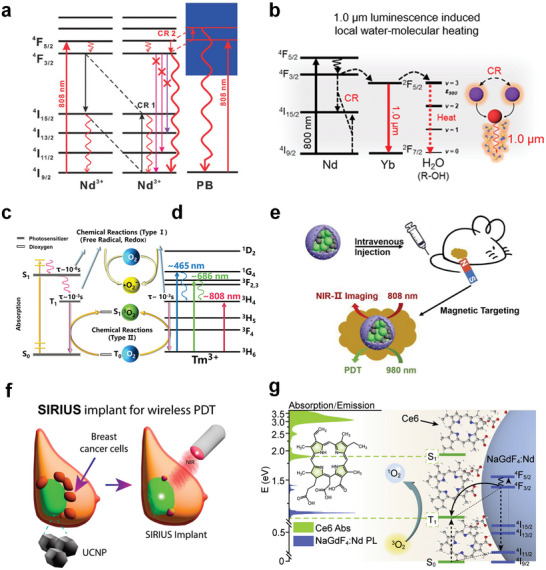
NIR‐Responsive RENPs for PTT and PDT. a) Simplified diagrams illustrating the generation of new CR pathways between Nd^3+^ and PB. Reproduced with permission.^[^ ^137^ ^]^ Copyright 2019, Wiley‐VCH. b) Illustrations of CR‐induced 1000 nm NIR emissions heating an aqueous solution. Reproduced under terms of the CC‐BY license.^[^ ^32^ ^]^ Copyright 2023, Springer Nature. c) Photochemical process of the photosensitizer. d) Proposed mechanism for ROS generation using Tm^3+^. c,d) Reproduced with permission.^[^ ^33^ ^]^ Copyright 2022, American Chemical Society. e) Schematic illustration of magnetically targeted NIR‐II bioimaging and PDT in mice. Reproduced under terms of the CC‐BY license.^[^ ^159^ ^]^ Copyright 2023, Wiley‐VCH. f) Schematic illustration of SIRIUS implant for wireless PDT. Reproduced with permission.^[^ ^161^ ^]^ Copyright 2023, American Chemical Society. g) Schematic illustration of the proposed direct triplet sensitization process in a NaGdF_4_:Nd^3+^‐Ce6 hybrid system. Reproduced with permission.^[^ ^167^ ^]^ Copyright 2021, Elsevier.

Nd^3+^‐doped nanoparticles have demonstrated good performances as emerging photothermal agents.^[^ ^91^, ^135^, ^136^ ^]^ Upon excitation by 808 nm NIR light, they increase the population of the ^4^I_15/2_ state through CR (^4^F_5/2_ + ^4^I_9/2_ → 2 ^4^I_15/2_) and then realize photothermal conversion via an irradiative transition back to the ground state ^4^I_9/2_. As this process relies on CR between the same or neighboring Nd^3+^ ions, its photothermal performance is closely related to the Nd^3+^ doping concentration. However, concentration modulation plays a limited role in performance enhancement. Even highly Nd^3+^‐doped photothermal agents require a high excitation power density to reach the threshold temperature for the ablation of cancer cells. The construction of heterostructures is an excellent strategy for addressing this limitation. Yu et al. coated a layer of Prussian blue (PB) on Nd^3+^‐based nanoparticles to construct a NaNdF_4_@PB composite core‐shell structure.^[^ ^137^ ^]^ This heterostructure demonstrated superior performance compared to its individual components. Its photothermal conversion efficiency (60.8%) was significantly higher than that of NaNdF_4_ (8.7%) and PB (19.8%). This promising performance primarily stems from the formation of a new CR pathway (CR2) between Nd^3+^ and PB. This exponentially increases the population of Nd^3+^ ions in the ^4^F_3/2_ energy level, which in turn enhances the CR1 process, suppresses the downward radiative transition to NaNdF_4_ high‐energy levels, and improves the heat‐production capability of the heterostructure (**Figure** **4**a). In addition, the presence of the CR2 pathway makes the wavelengths of the photons absorbed by the PB closer to the maximum absorption band (690–720 nm) of PB. All aforementioned mechanisms contribute to the achievement of high photothermal conversion efficiencies. The performance of Nd^3+^‐based photothermal agents has been further augmented through the design of this class of composites, wherein Nd^3+^‐based nanoparticles with stepped energy levels are coupled with other materials possessing continuous energy bands. Wang et al. fabricated similar composites based on RENPs and PB.^[^ ^138^ ^]^ However, they used a NaErF_4_ core as a DSL imaging agent to achieve NIR‐II fluorescence imaging‐guided PTT. Guo et al. employed Nd^3+^‐doped photothermal agents as antibacterial agents against Escherichia coli.^[^ ^139^ ^]^ They used a yolk‐shell GOF:Nd^3+^, Yb^3+^, Er^3+^ composite, which served two functions: PTT and thermal sensing. This thermometer‐heater platform showed excellent photothermal conversion and sensitive luminescent thermometry (1.6% K^−1^ of maximum relative sensitivity). This system may be a feasible choice for achieving real‐time controlled PTT with high therapeutic accuracy. Additionally, the rational design and optimization of the core‐shell RENP structure can facilitate better photothermal treatment of tumors. Kang et al. developed a core‐shell nanoparticle (NaYF_4_:50% Yb^3+^, 2% Tm^3+^@NaYF_4_:40% Nd^3+^, 20% Yb^3+^) with an Nd^3+^ sensitizer for NIR fluorescence imaging‐mediated PTT of gliomas.^[^ ^32^ ^]^ Water and biological fluids exhibit strong absorption in the range of 950–1050 nm because water has high energy‐absorption coefficients in this range, owing to its O‐H stretching vibrations and overtones (Figure 4b). Although this renders nanomaterials responsive to the above wavelengths unsuitable for biological applications, judicious utilization of this feature is a sensible PTT strategy. RENPs help drugs cross the blood–brain barrier by targeting antibodies that modify the transmembrane protein CD133, which is preferentially expressed on glioma cell membranes. Furthermore, the sub‐millisecond‐scale NIR luminescence lifetime of Tm^3+^ emissions makes Tm well‐suited for realizing time‐gated imaging, distinguishing autofluorescence, and preventing the negative thermal effects of using continuous lasers on fluorescence imaging.

#### 3.2.3 NIR‐Responsive RENPs as Nanoplatforms to Construct Photothermal Agents

In addition to being photothermal agents, RENPs can assist other photothermal materials in enhancing absorption and improving the efficiency of photothermal conversion. Yang et al. designed a multi‐functional integrated nanoplatform for tumor diagnosis and treatment. The platform combined UCL imaging, MRI, and PTT, with gold nanorods and GdOF:Yb^3+^, Er^3+^ serving as the core and shell, respectively.^[^ ^140^ ^]^ The emission of UCNPs effectively overlaps with the absorption of gold nanorods. A fluorescence resonance energy transfer occurs between the two, and the system as a whole can convert visible‐NIR light into thermal energy. Moreover, doping with some rare earth ions has a modulating effect on the absorption spectral generation structure of photothermal agents. For example, the doping of Bi_2_Se_3_ with Pr^3+^ enhances the absorption efficiency of the photothermal agent in the NIR region owing to the rich 4f electronic structure of Pr^3+^. Moreover, it improves the resonance interactions between the dopant and matrix during NIR activation, which increases the photothermal conversion efficiency.^[^ ^141^ ^]^ Doping Er^3+^ into a WSe_2_ matrix results in a red shift in the Er absorption spectrum, which enhances the photothermal conversion efficiency under 808 nm NIR laser activation.^[^ ^142^ ^]^ By exploiting the above features, multifunctional diagnostic and therapeutic biomaterials based on RENPs can be designed and developed. In addition, researchers can utilize the strengths and features of RENPs and photothermal agents to achieve efficient imaging‐mediated therapies.

Several photothermal agents have been reported to form complexes with RENPs for tumor PTT, including PDA,^[^ ^143^ ^]^ Bi nanoparticles,^[^ ^144^ ^]^ and Cu_2−_ *_x_* S.^[^ ^145^ ^]^ However, these photothermal agents can directly absorb NIR light, and RENPs mainly serve as bioimaging agents. They are vital to the research on PTT and RENP applications in phototherapy. Here, we focused on the contribution of RENPs to achieving NIR‐light excitation of photothermal agents, in other words, their indispensable role in PTT and other phototherapies. Therefore, imaging‐mediated PTT has not been discussed in detail.

### 3.3 Photodynamic Therapy

#### 3.3.1 Mechanism of PDT

PDT has been officially used since 1976 when Kelly and Snell used hematoporphyrin derivatives to successfully treat bladder cancer.^[^ ^146^ ^]^ This is a non‐invasive treatment method that generates reactive oxygen species (ROS) by activating photosensitizers using specific light wavelengths to selectively kill tumor cells.^[^ ^147^, ^148^, ^149^ ^]^ It has the benefits of high tumor‐cell specificity, low recurrence rate, and minimal or non‐invasiveness in healthy tissues. Following decades of development, hundreds of photosensitizers have become available, some of which are used in clinical applications. Photosensitizers primarily consist of two major categories: inorganic (e.g., inorganic semiconductor materials) and organic photosensitizers (porphyrin, phthalocyanine, chlorin, tetrapyrrole, BODIPY, etc.).^[^ ^150^, ^151^, ^152^ ^]^

PDT kills tumor cells mainly via three main interrelated mechanisms: direct cytotoxic effects, indirect damage to the vascular system, and the induction of an inflammatory response that can activate the immune system.^[^ ^153^ ^]^ Generally, under light excitation at appropriate wavelengths or matched energies, photosensitizers are first excited to the singlet excited state and subsequently populate the triplet state via an intersystem crossing (ISC) mechanism (S_1_→T*_n_*; *n* ≥ 1). Therefore, effective ISC is a crucial factor influencing photochemical applications. The long‐lived triplet excited states can transfer energy to other molecules to generate ROS (^1^O_2_, ·OH, and O_2_^*−^) via type I or type II reactions for tumor‐cell killing.^[^ ^154^, ^155^ ^]^ Type I reactions involve the production of free radicals as a result of electron transfer, whereas type II reactions excite molecular oxygen (^3^O_2_) into the highly reactive singlet oxygen (^1^O_2_) (Figure 4c). Among the various ROS, ·OH is relatively more toxic, and type I reactions are more advantageous in microenvironments with low molecular oxygen concentrations.^[^ ^156^, ^157^ ^]^ The half‐life of ^1^O_2_ is slightly longer, reaching the microsecond scale.^[^ ^158^ ^]^

#### 3.3.2 NIR‐Responsive RENPs as Photodynamic Agents

The excitation‐light source is a key factor affecting the effectiveness of PDT. The short‐wavelength region of UV–vis light is not ideal for deep tumor therapy because of its limited penetration depth, making the development of NIR‐responsive photosensitizers particularly important. In a recent study, Tm_2_O_3_ was shown to stimulate ROS production under NIR light activation owing to the large absorption cross‐section of Tm^3+^ at 808 nm and the long lifetime of the ^3^H_4_ excited state (Figure 4d).^[^ ^33^ ^]^ The successful use of Tm_2_O_3_ in tumor therapy opens new avenues for the biological applications of RENPs and indicates that RENPs play an increasingly important role in this field. Unfortunately, the use of RENPs as photodynamic agents has not been widely reported. Hence, researchers need to investigate this subject in detail and expand the material library.

#### 3.3.3 NIR‐Responsive RENPs as Nanoplatforms to Construct Photodynamic Agents

Most photosensitizers respond only to the UV–vis region, which hinders their use in cancer therapy. The excellent photoconversion ability of RENPs enables the activation of RENP‐based photosensitizers using NIR light. In a previous study, we used the microemulsion method to modularly assemble UCNPs with Fe_3_O_4_ nanoparticles, which were coated with mesoporous silica (mSiO_2_) and loaded with the photosensitizer zinc phthalocyanine (ZnPc), to construct nanocomposites facilitating magnetically targeted NIR‐II imaging guidance for PDT (Figure 4e).^[^ ^159^ ^]^ These nanocomposites exhibit a high degree of accumulation on tumor cells in response to an applied magnetic field. The ZnPc absorbs light in the visible region, which is highly matched with the upconversion emission under 980 nm excitation. Therefore, PDT was realized under NIR excitation. Moreover, the diagnostic and therapeutic agents were activated separately on demand and guided by NIR‐II imaging under 808 nm excitation. Here, ZnPc serves both as a photosensitizer and as a fluorescence imaging contrast agent, which is an intriguing finding. Cai et al. designed a core‐shell smart nanostructure based on UCNPs and the photosensitizer methylene blue (MB): UCNPs/MB@ZIF‐8@catalase.^[^ ^160^ ^]^ Catalase catalyzed the decomposition of overexpressed H_2_O_2_ in a tumor microenvironment to produce O_2_, thereby overcoming the lack of an oxidative environment at the tumor site. The researchers used a ZIF‐8 shell, which prevented the aggregation and leakage of MB molecules and increased the efficiency of Förster resonance energy transfer (FRET) from the UCNPs to MB to 17.9%, promoting the generation of ^1^O_2_ and enhancing the PDT effect. Considering the degradability and biosafety issues, Zhang et al. designed a flexible upconversion implant named “SIRIUS” (Figure 4f).^[^ ^161^ ^]^ The presence of the photosensitizer 5‐aminolevulinic acid (5‐ALA) enables the implant to realize PDT. 5‐ALA is a clinically established dye with a well‐documented safety record. SIRIUS demonstrated excellent therapeutic efficacy in rodent models of in situ breast cancer and may play an important role in the future clinical treatment of human cancers. The organic photosensitizers chlorin e6 (Ce6),^[^ ^162^ ^]^ hypericin (Hyp),^[^ ^163^ ^]^ and rose Bengal (RB)^[^ ^164^ ^]^ and the inorganic photosensitizers CeO_2_^[^ ^165^ ^]^ and TiO_2_^[^ ^166^ ^]^ can also realize NIR‐activated PDT based on the above principles; that is, the emission of RENPs under NIR excitation is utilized to effectively activate the photodynamic agents.

As mentioned, common UCNP‐photosensitizer composite systems excite photosensitizers from the singlet ground state to the triplet excited state via FRET using the spectral overlap between the upconversion emission and photosensitizer absorption. Unlike previous studies, Zheng et al. proposed a novel NIR photosensitization strategy for direct Ln triplet‐state energy transfer using NaGdF_4_:Nd^3+^‐Ce6 as a proof‐of‐concept example (Figure 4g).^[^ ^167^ ^]^ This direct sensitization system significantly reduced the loss of energy during the transfer process, and its efficiency in generating ^1^O_2_ was two orders of magnitude higher than that of the traditional FRET system. The system achieved a remarkable degree of tumor killing under NIR light activation with a low power density of 80 mW cm^−2^. The design allows the photosensitizer to produce ROS under low NIR irradiation, which improves its performance by more than hundred times compared to that of conventional UCNP‐based NIR photosensitization and may provide new opportunities for applications such as deep tumor phototherapy and NIR light‐driven photosynthesis.

PDT, which has garnered significant attention in oncological treatments, has also shown promising results in treating other diseases. Liao et al. designed functionalized UCNPs for the treatment of tuberculosis (TB).^[^ ^168^ ^]^ They loaded the photosensitizer pyrolipid (a lipid conjugate of pyropheophorbide‐a) and the anti‐TB drug rifampin onto the surface of the UCNPs with a mixture of lipids. The UNCPs converted NIR light to UV‐blue light, which was used to activate the photosensitizer and generate ROS to kill the surrounding TB bacteria. In addition, the rifampin‐induced cascade chemotherapy to combat bacteria that remained after PDT. This dual treatment showcased an outstanding performance and had a potent impact on the lesion. Refractory keratitis is another highly concerning disease. Zhang et al. combined aggregation‐induced emission photosensitizers and UCNPs, loaded NO donor, and generated ·O_2_^−^ and the highly toxic reactive nitrogen species, ONOO^−^. The system exhibited excellent anti‐bacterial and anti‐inflammatory properties.^[^ ^169^ ^]^ Chen et al. designed a Pt‐modified UCNP‐Zr‐MOF composite hydrogel that exhibited good biocompatibility.^[^ ^170^ ^]^ The Pt nanoparticles were employed as nanozymes to catalyze the decomposition of endogenous H_2_O_2_ into O_2_ for treating hypoxia. The nanoparticles thus significantly enhanced the photodynamic antibacterial efficacy of the Zr‐MOF, leading to accelerated wound healing. These reports illustrate that NIR‐responsive RENPs can be utilized to treat various diseases with broad application options.

### 3.4 Photoimmunotherapy

#### 3.4.1 Mechanism of PIT

Recently, immunotherapy has attracted the attention of researchers because of its high specificity, few side effects, and strong durability.^[^ ^171^, ^172^, ^173^ ^]^ PIT is the triggering of immunogenic cell death (ICD) in conjunction with light‐responsive therapy. In other words, it initiates an antitumor immune response causing a light‐mediated death of tumor cells. This follows a shift from a non‐immunogenic to an immunogenic state.^[^ ^41^, ^174^, ^175^ ^]^ Unlike traditional therapy, immunotherapy mobilizes the body's immune cells to produce a tumor‐specific immune response, prompting them to inhibit and kill tumor cells and motivate the body's immune system to “combat” tumors. Notably, this treatment approach can induce immunological memory and can play a long‐term role in impeding tumor recurrence and inhibiting metastasis to other tissues or organs.

#### 3.4.2 NIR‐Responsive RENPs as Nanoplatforms to Construct PIT Agents

In 1983, Levy et al. introduced the concept of PIT, documenting the term for the first time.^[^ ^176^ ^]^ In 2011, Mitsunaga et al. developed PIT further when they developed a targeted PIT agent based on the coupling of a monoclonal antibody with an NIR‐responsive cyanine dye, IR700.^[^ ^177^ ^]^ In 2020, Akalux, the first PIT drug in the world, was approved for clinical use in Japan.^[^ ^178^ ^]^ PIT has been hailed as the fifth most effective cancer treatment technology, following surgery, radiotherapy, chemotherapy, and cancer immunotherapy drugs. Akalux is an antibody‐coupled drug consisting of cetuximab and IR700, and it is used for the treatment of unresectable locally advanced or recurrent head and neck cancers. IR‐700 exhibits a high absorption coefficient, good water solubility, and low toxicity. Furthermore, the use of NIR light as an excitation source offers the advantages of low autofluorescence, deep tissue penetration, and weak tissue scattering.

**Figure 5:**
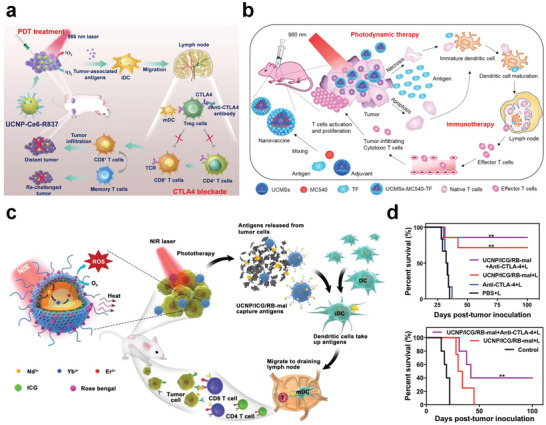
NIR‐Responsive RENPs for PIT. a) Scheme summarizing the mechanisms of combining NIR‐mediated PDT with CTLA‐4 checkpoint blockade for cancer PIT. Reproduced with permission.^[^ ^179^ ^]^ Copyright 2017, American Chemical Society. b) Schematic illustration of fabrication and mechanism of UCMSs‐MC540‐TF vaccines for PIT. Reproduced with permission.^[^ ^180^ ^]^ Copyright 2018, Wiley‐VCH. c) Schematic illustration of both fabrication and mechanism of NIR‐triggered antigen‐capturing nanoplatform for PIT. d) Survival curves of different groups of mice bearing orthotopic 4T1 tumors after different treatments (top, *n* = 6, **p < 0.01 vs control group) and survival rates after 4T1 tumor cell rechallenge in the mice (bottom, *n* = 4, **p < 0.01 vs control group). Data are expressed as mean ± SD. c,d) Reproduced under terms of the CC‐BY license.^[^ ^181^ ^]^ Copyright 2019, Wiley‐VCH.

However, the direct use of RENPs as PIT agents has rarely been reported. Currently, RENPs are mainly used as nanoplatforms for constructing PIT agents in combination with other PIT materials. Fortunately, RENPs can serve as PIT agents with a response range in the UV–vis region, enabling them to respond to NIR excitation. Liu et al. loaded Ce6 and imiquimod (R837), a toll‐like receptor‐7 agonist, onto UCNPs and constructed a rare‐earth nanoplatform for triggering antitumor immunity via PDT (**Figure** **5**a).^[^ ^179^ ^]^ By introducing a cytotoxic T‐lymphocyte‐associated protein 4 (CTLA‐4) blocker, the activity of the T_reg_ cells can be effectively inhibited, and the immunosuppressive environment within the tumor cells can be regulated. The proposed system treated the primary tumor thoroughly. The distant tumor model showed that the spread of the tumor cells was significantly inhibited. Notably, the induced immunological memory prevented a recurrence of the tumor. This study effectively investigated the potential of involving UCNPs in PIT and demonstrated the considerable potential of UCNPs for various applications. Similarly, Ding et al. obtained successful PIT results in CT26 hormonal BALB/c mice implanted with colon cancer cells (Figure 5b).^[^ ^180^ ^]^ They utilized macroporous silica‐modified UCNPs as a therapeutic platform to transport the photosensitizer merocyanine 540, model protein ovalbumin, and responsive tumor cell fragments (TFs). NIR‐activated ROS kill tumor cells, and their fragments act as antigens to stimulate the maturation of dendritic cells, which is enhanced by exogenously introduced TFs. Effector T cells are released from the lymph nodes and the antitumor immunity of the body is activated. The activation and proliferation of T cells, as well as the release of related cytokines, can significantly kill the tumor cells. By leveraging the remarkable photoconversion ability of the UCNPs, the loading capacity of the macroporous carriers, and cooperation between the photosensitizers and immunoadjuvants, this study exploited the potential of the various components to the fullest. Further, it provided deeper insights and references for PIT and directions related to immune vaccine delivery. Chen et al. designed a UCNPs/indocyanine green (ICG)/RB‐mal nanocomposite, which is another classic example of phototherapy‐induced ICD (Figure 5c).^[^ ^181^ ^]^ It was used in conjunction with a CTLA‐4 blocker in 4T1‐hormonal mice injected with poorly immunogenic and highly metastatic breast cancer cells, producing a long‐term (100 days) survival rate of ≈84%. Approximately 34% of the mice acquired tumor‐specific immunity (Figure 5d). The proposed system induced a strong long‐term immune memory, which protected the treated mice from tumor recurrence. This study offers a new strategy for improving therapeutic outcomes and inhibiting metastatic tumor relapses.

### 3.5 Optogenetics

#### 3.5.1 Mechanism of Optogenetics

Optogenetics is a revolutionary interdisciplinary technology that integrates optical and genetic techniques. It targets and introduces suitable exogenous light‐sensitive proteins into living cells via genetic methods. The light‐sensitive proteins are then stimulated using specific wavelengths of light, thereby regulating neuronal activity and controlling the changes in cellular and animal behaviors.^[^ ^5^, ^182^ ^]^ Compared to traditional methods, optogenetics has several advantages. It requires only the transfer of a protein into the cell and is highly practical. Using light as a stimulation medium allows millisecond manipulation of neurons. Compared to conventional methods, it involves considerably less harm to the experimental animals and does not entail any foreign‐body intrusion into the body tissues. Optical fibers can be employed to stimulate cells locally or scattered light can be designed to achieve widespread stimulation.

In a typical optogenetic regulatory process, a viral vector is first used to transmit a photoreceptor gene into a specific neuron to express a specific ion channel or G protein‐coupled receptor in the neuron.^[^ ^183^, ^184^ ^]^ When the neuron is in a resting state, a potential difference exists between the two sides of its cell membrane, known as the resting potential. The resting potential is generated by the uneven distribution of various ions inside and outside the membrane, as well as the different permeabilities of the cell membrane to various ions. The photoreceptor ion channels in the membrane selectively pass cations (e.g., H^+^, Na^+^, K^+^, and Ca^2+^) or anions (such as Cl^−^) through the membrane, under the stimulation of different wavelengths of light. This changes the membrane potential on both sides of the cell membrane and achieves selective excitation or inhibition of the cell.^[^ ^41^, ^185^, ^186^ ^]^ The exogenous stimulating light must have an excellent tissue penetration capability. However, this is challenging to achieve, as the absorption range of the photoreceptor genes or photoreceptor ion channels is mainly concentrated in the visible region. Hence, RENPs can utilize their excellent photoconversion ability to convert NIR light, which can penetrate deep tissues, into visible light emitted at the desired wavelength, enabling efficient optogenetic modulation.

#### 3.5.2 NIR‐Responsive RENPs for Optogenetics

**Figure 6:**
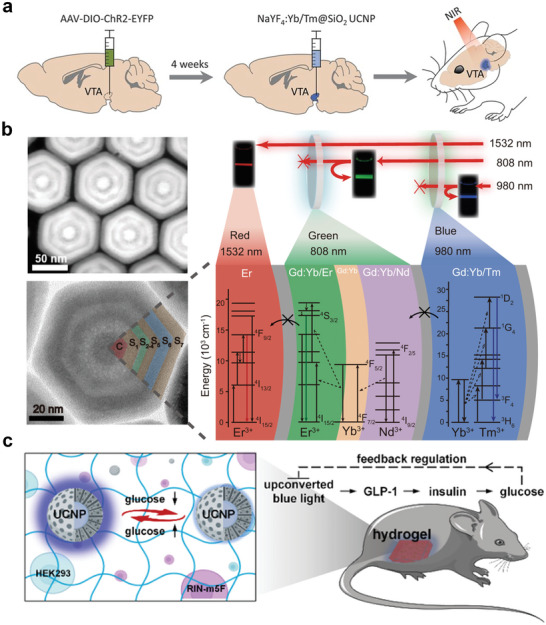
NIR‐Responsive RENPs for optogenetics. a) In vivo experimental scheme for transcranial NIR stimulation of the VTA in anesthetized mice. Reproduced with permission.^[^ ^193^ ^]^ Copyright 2018, The American Association for the Advancement of Science. b) The high‐angle annular dark field scanning transmission electron microscopy image (top‐left) and high‐resolution transmission electron microscopy image (bottom‐left) of the obtained multilayer UCNPs and the corresponding schematic illustration of energy dissipation upconversion process in multilayer UCNPs. Reproduced under terms of the CC‐BY license.^[^ ^194^ ^]^ Copyright 2021, Springer Nature. c) Schematic illustration of blood glucose reversibly modulated upconversion nanoprobes and closed‐loop glycemic control. Reproduced with permission.^[^ ^196^ ^]^ Copyright 2023, American Chemical Society.

In 2015, two articles published around the same time by Yawo et al. and Lee et al. linked UCNPs to optogenetics.^[^ ^187^, ^188^ ^]^ They utilized the UCL of Er^3+^ and Tm^3+^ in the visible region for the optogenetic activation of neurons inserted with a photoreceptor ion channel, rhodopsin (ChR), to modulate the membrane potential. The two studies not only extended the light response to the NIR region but also provided new insights into in vivo deep‐tissue optogenetics. A surge in optogenetic studies has been observed since the studies were published. Researchers have established biological models, such as Caenorhabditis elegans,^[^ ^189^, ^190^ ^]^ zebrafish,^[^ ^191^ ^]^ and the brain tissues of mammalian animals,^[^ ^192^ ^]^ for in vivo optogenetic investigations. McHugh et al. injected Tm^3+^‐doped nanoparticles into the ventral tegmental area (VTA) of a mouse for NIR‐responsive optogenetic studies.^[^ ^193^ ^]^ They created transgenic mice expressing tyrosine‐hydroxylase‐driven cyclization recombination enzyme (Cre) and introduced ChR2‐enhanced yellow fluorescent protein (EYFP) to induce Cre‐dependent expression of ChR2 in dopamine (DA) neurons. The DA neurons were activated after extracranial 980 nm NIR irradiation (**Figure** **6**a). In this system, neuronal excitation in the ChR2‐transfected mice is controlled only using NIR light. In addition, the report described a system incorporating green‐light‐activated archaerhodopsin (Arch)‐EYFP and Er^3+^‐based UCNPs, which expands the literature on NIR‐responsive optogenetics.

The field of UCNP‐based NIR‐responsive optogenetics is continuously evolving. Recent studies have proposed novel angles and perspectives in this field, which have enriched the depth of NIR‐responsive optogenetics research and laid the foundation for future biological applications. Zhang et al. designed trichromatic UCNPs excited by three wavelengths. The UNCPs achieved characteristic visible‐light emissions at 450 nm (blue), 540 nm (green), and 650 nm (red) under NIR‐light activation at 808, 980, and 1532 nm, respectively (Figure 6b).^[^ ^194^ ^]^ The spectral line widths of trichromatic UCL materials are narrow, which enables their precise modulation. They created parvalbumin (PV)‐Cre: somatostatin (SOM)‐flipase (flp) transgenic mice, wherein three different types of proteins, PV, SOM, and calcium/calmodulin‐dependent protein kinase II (CaMK II), were expressed in the mouse cortical neurons. The three optogenetic proteins were activated by red, green, and blue light. After the injection of the trichromatic UCNPs, the three types of neurons were activated separately by employing three types of NIR light to transcranially and selectively modulate the speed of living mice. Chu et al. developed an implantable wireless energy conversion device by loading UCNPs onto a gold inverse opaline skeleton grown with a dendrite‐like gold nanostructure.^[^ ^195^ ^]^ By utilizing the synergy between the photonic crystal effect and localized surface plasmon resonance (LSPR), the system allows for high UCL efficiency at low‐power NIR excitation to achieve implantable neuronal communication. The device exhibited excellent flexibility and maintained its original UCL intensity under multiple bending cycles in different bending states. It was implanted into the sciatic peripheral nerves of C57BL/6 mice expressing ChR2, and NIR modulation of the compound muscle action potential was successfully achieved. Lu et al. developed a reversible bioprobe based on UCNPs and the hydrophobic regulation of energy transfer mechanisms for in situ real‐time monitoring of blood glucose levels (Figure 6c).^[^ ^196^ ^]^ Glucose‐concentration‐dependent blue light effectively activated engineered human embryonic kidney 293 cells to secrete glucagon‐like peptide 1, which stimulated pancreatic islet cells to produce insulin for blood glucose control. The insulin‐induced reduction in blood glucose adjusts the luminescence intensity of the nanoprobes during the feedback process, which adaptively inhibits gene activation. Such glucose‐responsive nanoprobes provide a powerful tool for addressing the problem of optogenetic overtreatment and open a new window for synthetic biology‐based therapies.

The above studies suggest that the optogenetic regulation of RENPs is becoming increasingly important. The scope and complexity of the problems under investigation are expanding. The number of NIR‐responsive photoreceptor genes or photoreceptor proteins is limited. Therefore, the deep‐tissue penetration capability of NIR is required for this technique. Therefore, the value of NIR‐responsive RENPs in optogenetics is expected to steadily increase. Moreover, some researchers have been concerned about the non‐physiological activity patterns induced by the simultaneous action of various light stimuli on a population of neurons. This is not observed in NIR‐responsive RENP‐mediated optogenetics, where NIR light stimulation affects only the fraction of accumulated RENPs and does not significantly affect other neurons. In conclusion, the optogenetic applications of RENPs are promising.

### 3.6 Light‐Responsive Drug Release for Therapy

#### 3.6.1 Mechanism of Drug Release

Despite inducing some side effects, chemotherapy currently remains the major modality of cancer clinical treatment. In view of the current significance and irreplaceability of chemotherapeutic drugs, reducing the toxic side effects of chemotherapy and mitigating the indiscriminate killing of normal cells by chemotherapy drugs are some of the directions that must be investigated. For improving the targeting accuracy of drugs, controlled release of the drugs at a fixed point can facilitate their accumulation at the tumor site and prevent their leakage into normal tissues during blood circulation.^[^ ^197^, ^198^ ^]^ Stimulus‐responsive nanoparticles can significantly help in addressing this issue.

Stimulus‐responsive nanoparticles can sense endogenous or exogenous stimuli and respond accordingly. Exogenous stimuli mainly include external energy‐field stimuli such as sound, light, and heat, while endogenous stimuli consist of pH, the concentrations of H_2_O_2_ and glutathione in the tumor microenvironment, enzymes, and nucleic acids.^[^ ^199^, ^200^ ^]^ Responsive platforms can be constructed using associated responsive molecules or groups whose composition, structure, or conformation change in response to a stimulus. It can also target the release of prodrugs that are activated in response to specific stimuli.^[^ ^201^, ^202^ ^]^ In this section, we focus on controlled drug release achieved using light as an exogenous stimulus. This can be implemented in two ways: either light is used as the direct source of stimulation or drugs are released owing to light‐induced changes in the microenvironment. Light plays a vital role in both cases. Deep‐tissue‐penetrating NIR light proves to be highly effective for this purpose too.

#### 3.6.2 NIR‐Responsive RENPs for Controlled Drug Release

##### Direct Light‐Responsive Drug Release

RENPs are characterized by a high specific surface area. Furthermore, their surface enables easy modification and construction of heterostructures, making them excellent alternative carriers. Drugs can be encapsulated on the surface of RENPs using light‐sensitive molecules to avoid their early leakage over a long blood‐circulation time and realize site‐specific spatiotemporally controlled drug release and local functional modulation.^[^ ^5^, ^172^, ^203^ ^]^ In conjunction with the excellent photoconversion capabilities of RENPs, NIR light can activate the controlled release of drugs and other functional biomolecules. Several light‐sensitive molecules are available, and they are effective in a broadband spectral range, from UV to NIR. Furthermore, UCL and NIR‐II emission of Ln^3+^ can be used to realize fluorescence labeling of drug molecules, which enables the detection and tracking of drug delivery in real‐time without using other contrast agents.^[^ ^42^, ^204^, ^205^ ^]^

**Figure 7:**
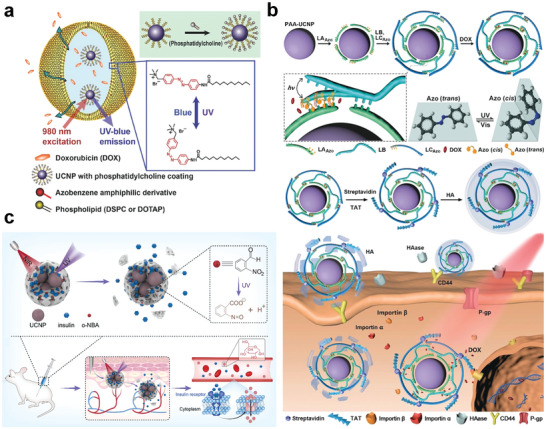
NIR‐Responsive RENPs for controlled drug release. a) Schematic illustration of NIR‐responsive azo‐liposome/UCNPs hybrid vesicles for controlled drug delivery. Reproduced with permission.^[^ ^206^ ^]^ Copyright 2016, Wiley‐VCH. b) Schematic illustration of constructing UCL‐driven DNA–azo nanopump and NIR‐responsive drug release in living cells. Reproduced with permission.^[^ ^207^ ^]^ Copyright 2019, Wiley‐VCH. c) Schematic illustration of the structure of the ZIF‐8‐based delivery system and working principles of NIR‐responsive protein release. Reproduced with permission.^[^ ^215^ ^]^ Copyright 2023, Elsevier.

Yao et al. constructed liposome‐encapsulated drug delivery nanoplatforms based on UCNPs for the spatiotemporal‐specific controlled release of doxorubicin (DOX) using light‐sensitive azo molecules (**Figure** **7**a).^[^ ^206^ ^]^ Under 980 nm laser irradiation, the UV–vis emission of Tm^3+^ activates the reversible isomerization of the azo molecules in the liposome framework. Azo trans‐isomers are converted to cis‐isomers under UV light, and the cis‐isomers are converted back to the trans‐isomers under visible light. This results in a continuous rotation‐reversal process in the liposomal membrane, thereby realizing controlled drug release. The amount and rate of release were determined according to the power density and duration of NIR irradiation. This is a significant advancement in the field of NIR‐responsive spatiotemporal‐specific drug release. The study provides new inspirations for drug delivery and a valuable reference for attenuating the side effects of chemotherapy. Zhang et al. designed a UCL‐driven DNA‐azo nanopump for targeted and controlled release of DOX (Figure 7b).^[^ ^207^ ^]^ The UV–vis emission from UCL under NIR excitation stimulates the sequential photoisomerization of azo, which acts as an impeller pump that triggers the cyclic hybridization and dehybridization of DNA. In addition to the above systems, light‐responsive drug release strategies based on UCNPs have been successfully employed for the photosensitive molecule 4‐(hydroxymethyl)−3‐nitrobenzoic acid,^[^ ^208^, ^209^ ^]^ a Ru‐complex‐based enzyme inhibition system,^[^ ^210^ ^]^ the chemotherapy drug blebbistatin,^[^ ^211^ ^]^ CO/NO donors,^[^ ^212^, ^213^ ^]^ and messenger RNA (mRNA).^[^ ^214^ ^]^ These reports have focused on cases where light acts directly on responsive molecules or groups, in coordination with the excellent photoconversion ability of RENPs, achieving controlled drug release and mitigating side effects and toxicity to normal tissues.

##### Light Modulation of Microenvironment for Drug Release

Light can also play a role in regulating the tumor microenvironment, which in turn promotes drug release. Li et al. constructed a NIR remote‐controlled drug delivery system using a self‐assembly strategy to achieve spatiotemporally controllable protein delivery and release (Figure 7c).^[^ ^215^ ^]^ The authors encapsulated UCNPs and proteins inside ZIF‐8 and introduced photosensitive acid‐producing agents into the ZIF‐8 pores. UV emission under 980‐laser irradiation selectively stimulates the release of protons from the acid‐producing agents, causing a local pH decrease to trigger ZIF‐8 degradation and thus realizing a controlled release of the proteins. Using insulin as a protein model, researchers have validated the feasibility of remote in vivo regulation of blood‐glucose concentration using NIR, which can penetrate deep into tissues and maintain a stable blood glucose concentration over a long time. This modular design concept provides a versatile and new approach for the on‐demand precise release of proteins. This type of drug release, which is mediated by the modulation of the microenvironment using light, expands the application of light in the field of drug delivery and controlled release. Currently, some studies have been conducted in this field, which has notably contributed to promoting the research on controlled drug release.

### 3.7 All‐in‐One Therapy

In view of the complexity, diversity, and heterogeneity of malignant diseases, their treatment may often be inevitably hindered by the limitations of the therapy method employed; therefore, in many cases, a combination of multiple treatment modalities must be realized on a single nanoplatform. In addition, many treatment approaches are usually only effective for early‐stage cancers and not for late‐stage diseases. Diagnosis at the earliest possible stage can maximize the treatment effectiveness, and precise imaging monitoring during the treatment process can facilitate efficient therapy. Therefore, diagnostic and therapeutic multifunctional platforms must be developed.^[^ ^216^, ^217^, ^218^ ^]^ Theranostics is currently a popular research topic.

Light often performs multiple functions in practical applications. Researchers want to synergize associated treatments to achieve a coordinated action of multiple therapies in a single functional nanoparticle or easy‐to‐construct heterogeneous structures. As light is the initiator of the entire treatment, it must be re‐emphasized that light used for the treatment should exhibit excellent tissue penetration, low autofluorescence, and photon scattering. Frequently, multiple‐wavelength emissions are required to accommodate the respective wavelength responses of multiple units. All these problems can be addressed using RENPs, which can serve as the basis for building multifunctional nanoagents that can easily integrate multiple imaging and therapy modalities. This enables the precise diagnosis of lesions and efficient minimal or non‐invasive imaging‐guided therapy.

**Figure 8:**
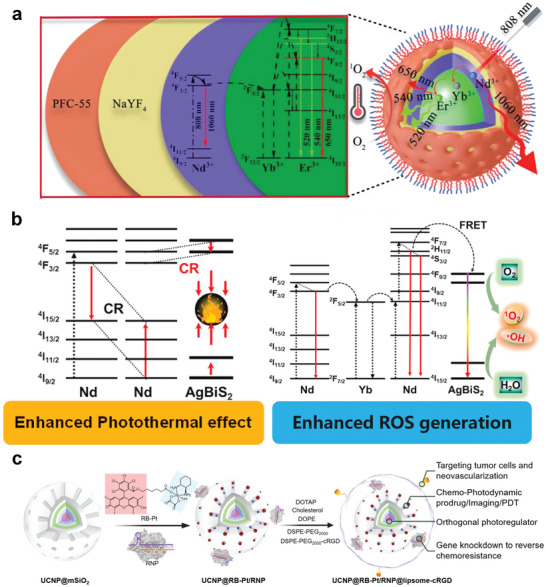
NIR‐Responsive RENPs for combined phototherapy. a) The structure and a possible mechanism for ROS generation of UCNPs/HOFs. Reproduced with permission.^[^ ^220^ ^]^ Copyright 2023, Elsevier. b) Schematic illustration of the generation of cross‐relaxation pathways between Nd^3+^ and AgBiS_2_ (left) and the enhanced generation of ROS between Er^3+^ and AgBiS_2_ (right). Reproduced with permission.^[^ ^223^ ^]^ Copyright 2022, Elsevier. c) The construction of nanodrugs for combined cancer phototherapy and description of the functions of every component. Reproduced with permission.^[^ ^229^ ^]^ Copyright 2023, Royal Society of Chemistry.

Liu et al. proposed an integrated theranostic nanomotor, UCNPs@mSiO_2_‐Au‐Cys (Cys = Cy‐S‐Ph‐NH_2_), to realize UCL/photothermal imaging‐guided PTT/PDT combined therapy.^[^ ^219^ ^]^ Au nanoparticles, as a photothermal agent, provide heat and act as a driving source for nanomotors because of their asymmetric distribution on the UCNPs. In addition, Au nanoparticles have been used as nanozymes for decomposing H_2_O_2_ into O_2_ to enhance the PDT effect of the photosensitizer Cys. The excellent luminescence properties of the UCNPs help the nanomotors achieve NIR‐imaging‐guided therapy and detect online ^1^O_2_ levels to reflect the efficiency of the tumor treatment. Our group coated UCNPs with a hydrogen‐bonded organic framework (HOF), PFC‐55, via a ligand‐grafting stepwise synthetic method and realized NIR‐II‐guided combined PTT and PDT on the same nanoplatform under 808 nm excitation.^[^ ^220^ ^]^ PFC‐55 has a wide absorption range in the visible region, which can be used for realizing Er^3+^ UCL. The proposed system exhibited excellent ^1^O_2_ generation and photothermal conversion ability under NIR light excitation (**Figure** **8**a). The emissions from Nd^3+^ at 1060 nm (^4^F_3/2_ → ^4^I_11/2_) can significantly help achieve real‐time NIR‐II imaging monitoring. This study is similar to previous related reports. For the first time, composites of UCNPs and HOFs have been applied to in vivo tumor therapy.^[^ ^221^ ^]^ This interesting research advancement broadens the material library on the functionalization of UCNP nanoplatforms for NIR‐II imaging‐guided in vivo tumor therapy. Wu et al. designed a ROS nanogenerator for tumor treatment by integrating a pH‐dissociable Fe^3+^–gallic‐acid complex shell with a Ce6‐coupled RENP photosensitizer core.^[^ ^222^ ^]^ They achieved NIR‐II/PA dual‐imaging‐guided PTT/PDT/ferroptosis therapy, which activated strong anti‐tumor immunity to effectively inhibit tumor metastasis, providing a paradigm for imaging‐guided cancer treatment. In another recent study, AgBiS_2_ was shown to achieve synergistic tumor therapy, which could be augmented using UCNPs.^[^ ^223^ ^]^ Owing to the CR between Nd^3+^ and AgBiS_2_, the photothermal conversion efficiency of AgBiS_2_ increased from 14.7% to 45% under 808 nm laser irradiation. The upconversion emission from Er^3+^ matches the energy band structure of AgBiS_2_, which can drive type I and type II PDT reactions to occur simultaneously. This produces ^1^O_2_ and ·OH for tumor therapy (Figure 8b). The proposed composite can also be used as an antibacterial agent against methicillin‐resistant *Staphylococcus aureus*.^[^ ^224^ ^]^

As mentioned previously, PDT/PTT combination therapy and optical imaging for guidance and monitoring based on NIR‐responsive RENPs play an increasingly important role in wireless phototherapy for other diseases. Kataria et al. designed a sandwich structure by plasmonically coupling two different Au nanoparticles (AuNPs) with UCNPs using surface modification techniques.^[^ ^225^ ^]^ Owing to LSPR, the UCL was dramatically enhanced, and the high‐intensity green fluorescence rendered the composite viable a choice for cellular bioimaging. The two AuNPs exhibited PTT and PDT antibacterial activities. Zhou et al. developed a highly effective photosensitizer,^[^ ^226^ ^]^ poly(selenoviologen), which self‐assembled on the surface of UCNPs and killed methicillin‐resistant *S. aureus* under mild laser irradiation conditions (150 mW cm^−2^ for 4 min). This study implemented a synergistic phototherapy system triggered by low‐power single‐wavelength NIR light, providing a simple and versatile strategy for developing safe and clinically translatable agents for the effective treatment of deep tissue bacterial inflammation. Gao et al. synthesized a vasoactive intestinal peptide‐loaded nanocomposite, UCNPs@SiO_2_@Pt@CeO_2_, for treating rheumatoid arthritis (RA).^[^ ^227^ ^]^ New directions of RA treatment involve relieving hypoxia and removing synovial cells. Correspondingly, CeO_2_ decomposes H_2_O_2_ to produce O_2_ in situ to relieve hypoxia and catalyze ROS generation using NIR light and UCNPs to inhibit the proliferation of fibroblast‐like synovial cells. Additionally, Pt nanoparticles are good photothermal agents and Pt‐based photothermal imaging demonstrates their excellent in vivo properties directly. This multifunctional nanoplatform integrates in situ O_2_ generation, PDT, and PTT and expounds its potential as a new treatment strategy for RA.

Additionally, light‐responsive drug release can be integrated with other phototherapy methods. Li et al. designed a multilevel shell structure, UCNPs‐mSiO_2_‐Ce6‐Se‐Se‐mSiO_2_‐DOX, for realizing synergistic chemotherapy and PDT.^[^ ^228^ ^]^ The di‐selenide‐bridged structure easily responds to ROS; therefore, the Ce6‐mediated PDT disintegrates the Se‐Se‐mSiO_2_ shell and releases DOX rapidly. UCL also plays a role in fluorescence imaging for monitoring the delivery of nanoplatforms. This is a typical example of an NIR‐responsive RENP that integrates diagnostic and therapeutic functions. Liu et al. graded controlled functional blocks for synergistic therapy using orthogonal emissions from UCNPs (Figure 8c).^[^ ^229^ ^]^ Ho^3+^ emission under 980 nm excitation activated Pt (IV) prodrugs. The emitted photons were absorbed by an RB photosensitizer for PDT. Tm^3+^ emission under 808 nm irradiation activated the gene‐editing tool Cas13d, which attenuated the tumor resistance and induced large‐scale targeted‐drug accumulation. This leads to an effective eradication of the primary tumors and successful inhibition of liver metastasis. This study introduces binary NIR light‐responsive nanoprodrugs that can orthogonally control tumor treatment. The development of the genome editing toolbox also complements the expansion of RENP applications effectively.

Tumors are characterized by their diversity, complexity, and heterogeneity. Owing to the above, the effectiveness of a single treatment may be limited when tumors occur unexpectedly. Therefore, a synergistic therapy is imperative. RENPs can serve as a foundation for integrating the advantages of other materials to build multifunctional diagnostic and therapeutic integration platforms, synergizing multiple light‐mediated treatments and amplifying the effects of individual functional units as much as possible. This promising field has the potential to replace the traditional treatments. Therefore, researchers must strive to bridge the gap between the laboratory and clinical applications in this field.

## 4 Summary and Outlook

Considering the applications of in vivo optical imaging and wireless phototherapy, the light source employed must at least exhibit deep tissue penetration, low autofluorescence, and low tissue scattering. Currently, NIR light is undoubtedly a very suitable choice for the same. None of the AuNPs photothermal agents, ICG contrast agents, or Akalux PIT drugs currently in clinical trials or already in clinical use can be activated without using NIR light. Unfortunately, not all phototherapeutic agents can be triggered by NIR light and many photosensitive particles respond only to the UV–vis region. The use of NIR light is also often limited by the fixed narrow‐wavelength bands of commercially available NIR lasers, which do not provide the desired absorption region. All these factors heavily constrain the realization of efficient NIR phototherapy. Rare earth ions, with their rich 4f energy level structure, offer enough “space” for efficient light‐to‐light transformation and the conversion of light to other forms of energy. Recently, research on RENPs has progressed tremendously. However, this does not imply that complacency is justified: opportunities and challenges persist continually.

The most critical challenge faced by RENP UCL is its low luminescence efficiency, which becomes particularly pronounced for large anti‐Stokes shifts. Currently, UCL optimization strategies include the following three approaches. The first strategy is to consider an inert‐ion doped host matrix that can change the crystal field symmetry and reduce the resistance against the 4f–4f electric dipole forbidden transitions in Ln^3+^. The second strategy is to enhance the absorption of the excitation light by particles, which can be achieved using a sensitization strategy. Even Nd^3+^ and Yb^3+^, which have considerable absorption cross‐sections, fail to deliver the high‐intensity and high‐efficiency luminescence required for practical applications. Therefore, the use of sensitizers, such as organic dyes, that can extend the Ln^3+^ absorption cross‐section by several orders of magnitude is often necessary. The third strategy is to amplify the emitted light or reduce the quenching of active ions using an inert shell layer coating. The coating reduces the surface defects and isolates the sensitizers and activators from the quenching environment on the surface to decrease the loss during energy transfer. Several strategies have been developed that successfully improved the efficiency and intensity of UCL intensity to some extent. However, limited efficiency enhancement has been achieved with conventional fluorides and oxides. Existing well‐established methods do not permit further improvements. High efficiency is often obtained by severely limiting the test conditions. Therefore, the development of new enhancement tools for practical applications is necessary and urgent. The phonon energy of an intrinsic lattice is a significant constraint. The development of novel nanoscale luminescent substrates with low phonon energy is also a critical research area, which may help UCNPs achieve an intrinsically improved luminescence efficiency compared to conventional particles. The Stokes shift can be further increased for the DSL of NIR‐responsive RENPs. The downshifted emissions in the NIR‐IIc and NIR‐III regions should be emphasized. The DCL of NIR‐responsive RENPs has been reported previously. However, the concept is yet to be fully integrated into biological applications, requiring researchers to promote its development. NIR‐responsive PL materials are primarily UCPL materials combined with upconversion materials. The luminescent centers are excited by UV and visible light obtained from upconversion emission rather than by NIR‐light direct excitation. The energy transfer is accompanied by energy loss, which significantly reduces the luminescence efficiency. Therefore, the development of direct NIR‐activated PL materials may be crucial for the future of rare earth luminescence applications.

Biological agents for in vivo therapeutic applications must consider biosafety and biotoxicity. The biocompatibility of RENPs can be improved by modifying them with amphiphilic macromolecules, liposomes, peptides, nucleic acids, or biofilms. Ideally, the modification should lead to an improvement in targeting. The indiscriminate attack of nanodrugs on normal tissue cells cannot be ruled out. Relying solely on enhanced permeability and retention effects often proves ineffective, and targeting tumor cells using membrane‐specific receptors is a more promising strategy. Further, RENPs tend to aggregate in major organs, such as the liver and spleen. In some cases, they cannot be fully metabolized, even for weeks and months, which is potentially risky for organisms. Encapsulating RENPs within ultrasmall nanoparticles facilitates satisfactory metabolization of the RENPs through the hepatobiliary or renal pathways. However, in general, the surface defect density of ultrasmall nanoparticles is significantly higher than that of large particles, which severely affects the luminescence. In this situation, the control of luminescence may need to be revolutionized. Recently, several studies have been conducted on the use of degradable UCNPs for biological applications. Regrettably, they did not perform a systematic and comprehensive assessment as was necessary. This can be investigated further to prepare safer nanodrugs. However, rapid degradation of tumor cells may be undesirable; nanoparticles need a certain time to accumulate at the tumor site. The particles play a role in determining the spatiotemporal specificity required to initiate the therapeutic process and in completing short‐term metabolic tasks. This is a desirable state of affairs and a balance must be sought urgently.

Research efforts are aimed at narrowing the distance between basic research and clinical applications. Some metal nanoparticles have already advanced to the transition zone or achieved prominence in clinical applications. RENPs must not lag behind and strive to join these ranks as soon as possible. The selection of functional materials for optical imaging and phototherapy should follow the basic principles of safety, efficacy, and cost‐effectiveness required for clinical applications. Therefore, the development of novel materials should focus on the optimization and enhancement of the valuable systems reported in the past. These materials passed the initial test and must be further developed for their final application. In addition, historically, currently, and for the foreseeable future, the primary function of RENPs will continue to be photoconversion. However, this does not imply that the conversion of light into or out of other forms of energy can be ignored. On the contrary, light conversion is integral to the study of RENPs. Researchers must investigate the field of converting light to other forms of energy further and harness its potential more effectively in wireless phototherapy.

Overall, for future development, more efforts must be invested in the following aspects. Development of new methods to enhance the luminescence efficiency and intensity of RENPs and new matrices with lower phonon energies and nanosizes is required. The biocompatibility of the particles should be improved, and their targeting precision should be enhanced to prevent damage to normal tissues and cells. Therefore, the metabolism and harmful accumulation of these materials should be considered further. Stringent demands must be set for the study of long‐term toxicity and systemic toxicity of the organism. The development of light‐functional biomedical materials should consider the research and construction of new materials as well as the optimization and improvement of past materials while preserving the achievements and knowledge already gained. Accelerating the progress of RENP‐based treatment towards clinical applications of RENPs, with its outstanding advantages, is not only a necessity for researchers across relevant disciplines but also a collective expectation.

## Conflict of Interest

The authors declare no conflict of interest.
